# Sedation levels in dogs: a validation study

**DOI:** 10.1186/s12917-017-1027-2

**Published:** 2017-04-18

**Authors:** Marika C. Wagner, Kent G. Hecker, Daniel S. J. Pang

**Affiliations:** 1 Veterinary Clinical and Diagnostic Sciences, Faculty of Veterinary Medicine, University of Calgary, Calgary, AB Canada; 2 Départment de sciences cliniques, Faculté de medicine vétérinaire, Université de Montréal, Saint-Hyacinthe, Québec J2S 7C6 Canada; 3 Groupe de recherche en pharmacologie animale du Québec (GREPAQ), Faculté de medicine vétérinaire, Université de Montréal, Saint-Hyacinthe, Québec Canada

**Keywords:** Validity, Reliability, Canine, Dexmedetomidine, Anesthesia

## Abstract

**Background:**

The aim of this study was to assess validation evidence for a sedation scale for dogs. We hypothesized that the chosen sedation scale would be unreliable when used by different raters and show poor discrimination between sedation protocols.

A sedation scale (range 0–21) was used to score 62 dogs scheduled to receive sedation at two veterinary clinics in a prospective trial. Scores recorded by a single observer were used to assess internal consistency and construct validity of the scores. To assess inter-rater reliability, video-recordings of sedation assessment were randomized and blinded for viewing by 5 raters untrained in the scale. Videos were also edited to allow assessment of inter-rater reliability of an abbreviated scale (range 0–12) by 5 different raters.

**Results:**

Both sedation scales exhibited excellent internal consistency and very good inter-rater reliability (full scale, intraclass correlation coefficient [ICC_single_] = 0.95; abbreviated scale, ICC_single_ = 0.94). The full scale discriminated between the most common protocols: dexmedetomidine-hydromorphone (median [range] of sedation score, 11 [1–18], *n* = 20) and acepromazine-hydromorphone (5 [0–15], *n* = 36, *p* = 0.02).

**Conclusions:**

The hypothesis was rejected. Full and abbreviated scales showed excellent internal consistency and very good reliability between multiple untrained raters. The full scale differentiated between levels of sedation.

**Electronic supplementary material:**

The online version of this article (doi:10.1186/s12917-017-1027-2) contains supplementary material, which is available to authorized users.

## Background

Sedation describes a state where an animal’s response to external stimuli is reduced and sedating animals is a common procedure, used to improve safety during handling and to facilitate minor procedures without general anesthesia. Before general anesthesia, sedation is an important component of pre-anesthetic medication, providing anxiolysis, contributing to balanced anesthesia, potentially providing pre-emptive analgesia (if a sedative has analgesic properties) and producing a smooth recovery [1]. During trials for drug licensing and toxicology, the presence of sedation is monitored and recorded.

However, measurement scales for quantifying sedation in dogs have not been formally assessed for validity and reliability of the scores. Existing scales vary considerably in the number and content of scale items and scales are frequently altered between studies, impeding the direct comparison of sedation data between studies [2–8].

Assessing validation and reliability provides information on whether a scale measures what it claims to measure (validity) and the degree of measurement error (reliability). In the context of measuring sedation, establishing evidence for the validity and reliability of the scores are essential to ensure appropriate scale sensitivity when evaluating levels of sedation and acceptable agreement between raters. Furthermore, using an appropriately developed scale facilitates comparing results between studies, thereby supporting reproducibility.

The scale selected for evaluation was published by Grint et al. [3] in a study describing the sedative properties of the alpha_2_-adrenergic receptor agonist dexmedetomidine alone or in combination with the opioid meperidine to produce sedation in dogs. In reviewing the literature to trace the origin of the sedation scale evaluated in the study, it became apparent that the scale had evolved through numerous versions over approximately 27 years, with early versions having 4–5 items in common with the 7 items of the current scale, with varying ranges of scores for each item [2–10]. This evolution reflects iterative changes made by different authors but limits quantitative comparisons between studies: this acted as a stimulus to perform the study presented here.

The primary goal of this study was to gather evidence regarding the validity and reliability of the scores from the selected sedation scale by applying psychometric methods and, if the evidence was acceptable, to begin development of a simplified version of the scale. A secondary goal was to evaluate the psychometric performance of the full scale with a recently published scoring system for evaluating the quality of health measurement scale development [11].

We hypothesized that the scale would perform poorly, with a limited ability to detect differences between levels of sedation and show poor inter-rater reliability when used by multiple raters.

## Methods

### Animals

This project received ethics approval from the University of Calgary Veterinary Sciences Animal Care Committee (AC13–0103), which operates in accordance with Canadian Council on Animal Care guidelines. Dogs scheduled to be sedated for a diagnostic procedure or before general anesthesia were enrolled over a 12 week period through two clinics following written informed client consent. The choice of sedation protocol was at the discretion of the supervising veterinarian. Dogs were excluded if aggressive, had been given drugs with potential sedative effects earlier on the same day, were known to be deaf, or had an American Society of Anesthesiologists physical classification status >2. Dogs were housed individually in kennels in the clinic.

### Experimental procedure

A published scale was used to assess sedation at baseline (before drug injection) and 15 min after drug injection [3]. The scale included seven items: spontaneous posture, palpebral reflex, eye position, jaw & tongue relaxation, response to noise, resistance when laid into lateral recumbency, and general appearance/attitude (Table 1). Each item was assigned a score and scores summed to give a sedation score (range 0 to 21), with higher scores indicating a greater level of sedation. Response to noise was assessed with a clicker (i-Click Clicker, i-Click, Waltham, MA, USA) actuated approximately 150 cm from the head. For assessment of sedation each dog was taken to a quiet, empty room by the rater (MW). Assessments were also video-recorded (Hero 3+ GoPro camera, San Mateo, CA, USA).

**Table 1:** Sedation scale from Grint et al. [3]

1. Spontaneous posture	
• standing =0	
• tired but standing =1	
• lying but able to rise =2	
• lying but difficulty rising =3	
• unable to rise =4	
2. Palpebral reflex	
• brisk =0	
• slow but with full corneal sweep =1	
• slow but with only partial corneal sweep =2	
• absent =3	
3. Eye position	
• central =0	
• rotated forwards/downwards but not obscured by third eyelid =1	
• rotated forwards/downwards and obscured by third eyelid =2	
4. Jaw & tongue relaxation	
• normal jaw tone, strong gag reflex) = 0	
• reduced tone, but still moderate gag reflex =1	
• much reduced tone, slight gag reflex =2	
• loss of jaw tone and no gag reflex =3	
5. Response to noise (handclap)^a^	
• normal startle reaction (head turn towards noise/ cringe) = 0	
• reduced startle reaction (reduced head turn/ minimal cringe) = 1	
• minimal startle reaction =2	
• absent reaction =3	
6. Resistance when laid into lateral recumbency	
• much struggling, perhaps not allowing this position =0	
• some struggling, but allowing this position =1	
• minimal struggling/ permissive =2	
• no struggling = 3^b^	
7. General appearance/attitude	
• excitable =0	
• awake and normal =1	
• tranquil =2	
• stuporous =3	

^a^In this study, a clicker was used in place of a handclap in an effort to standardise the stimulus. Table reproduced with the written permission of the publisher, John Wiley and Sons, through the Copyright Clearance Centre

^b^A score of “2” was assigned in the published scale (Grint et al. [3]); this was confirmed as a typographical error with N Grint before beginning this study

All drugs used to provide sedation were combined into a single syringe and administered either intravenously (via cannula) or intramuscularly (lumbar epaxial muscles). Route of injection was at the discretion of the supervising veterinarian. Following injection, dogs were returned to their kennel and left undisturbed. Twelve minutes after drug injection dogs were walked or carried to the assessment room with a long (200 cm) leash attached and two minutes allowed for adjustment to surroundings so that assessments were performed 15 min after injection. The assessment room was a quiet, empty room (approximately 6 × 6 m) in which dogs were attached to the wall by the free end of the leash. The scale items were assessed in the same order each time: observation for spontaneous posture (at a distance of 150 cm), response to noise, eye position, palpebral reflex, jaw tone (and if reduced, a tongue depressor was placed at the base of the tongue to assess tongue relaxation and the swallow reflex), resistance when laid in lateral recumbency, and a final observation of general appearance/attitude. After completing the post-injection assessment, each dog continued along its intended care pathway (diagnostic procedure or general anesthesia).

Following data collection, a convenience sample of 15 videos was selected to represent different levels of sedation based on scores assigned by the primary rater (little/no sedation; score 0–2, moderate sedation; score 4–11, heavy sedation; score 13+, *n* = 5 videos per group). Videos were from 14 different dogs, including baseline (*n* = 4 videos) and post-injection (*n* = 11 videos) times. These were used to assess inter-rater reliability. Five registered animal health technologists voluntarily and independently watched each video and provided a sedation score using the published scale [3]. No instruction was given in use of the scale (none had previous experience of the scale) and raters were blinded to treatment and time point.

Finally, the sedation scale was simplified by removing items felt to be more invasive or stressful to the dogs, or potentially increasing risk to personnel. These were items 2, 4 and 6: palpebral reflex, jaw and tongue relaxation, and resistance when laid into lateral recumbency. Assessment of these items was edited out of each of the 15 videos. Edited videos were scored by five different technologists or interns using the simplified version of the scale (abbreviated scale range 0–12). The new raters were also blinded to treatment and time point, and had no previous experience with the scale.

### Statistical methods

Internal consistency, the degree to which scale items are inter-related, was determined by calculating Cronbach’s alpha of the scores assigned by the primary rater (MW). This was done for the full scale (7 items) and simplified scale (4 items) using post-injection scores. Internal consistency was considered excellent if Cronbach’s alpha was greater than 0.75 [12].

Inter-rater reliability, agreement between raters, was determined by calculating the intraclass correlation coefficient (ICC). The ICC was calculated for the full and simplified scale and classified as: very good (ICC 0.81–1.0), good (ICC 0.61–0.80), moderate (ICC 0.41–0.60), fair (ICC 0.21–0.40), poor (ICC < 0.20) [13, 14]. ICC data are presented for single (ICC_single_) and average (ICC_average_) measures, where ICC_single_ represents score reliability of a single rater performing a single observation and ICC_average_ represents score reliability of several raters or several observations. Both are presented as they indicate scale performance in different situations, though the more conservative ICC_single_ is likely to be a closer reflection of scale performance in typical use (single rater, single observation).

The efficacy of sedation protocols was assessed by comparing baseline with post-injection sedation scores with a Wilcoxon matched-pairs signed rank test. Sedation scores were compared between the two most common protocols with a Mann-Whitney test. Values of *p* < 0.05 were considered significant. Where available, the confidence interval (CI) of the mean or median difference between scores is reported. Analyses were performed with commercial software (IBM SPSS Statistics for Windows version 22.0, IBM Corp., Armonk, NY, USA and Prism v7.0, GraphPad Software, La Jolla, CA, USA). An overall assessment of the psychometric performance of the sedation scale was performed according to a recently developed scoring criteria for several different health measurement scales, including sedation (Appendix 1) [11, 15]. According to these criteria, scores are assigned to reflect assessment and reporting of validity, reliability and feasibility. A total score of ≥12/20 is considered to reflect acceptable psychometric properties. The relationship between scores assigned by the primary observer (MW) and untrained observers was explored by calculating a Spearman correlation coefficient for both the full and abbreviated scales (for the 15 videos analysed by all raters), with the median value of the 5 untrained observers used for calculation.

## Results

Seventy-five dogs were enrolled in the study. Thirteen dogs were excluded for: poor video quality or technical complications with recording equipment (*n* = 3), aggression (*n* = 2), failure to adhere to time points (*n* = 7) and additional sedation given during assessment period (*n* = 1). Sixty-two dogs were included in the analysis (Table 2).

**Table 2:** Demographic data for dogs from the two most common sedation protocols

Sedation protocol	Acepromazine - hydromorphone	Dexmedetomidine - hydromorphone
Number of dogs	36	20
Age (years)	3 (0.25–11)	3 (0.58–10)
Sex	18 females, 11 males, 6 spayed females, 1 neutered male	7 females, 6 spayed females, 4 neutered males, 3 males
Mass (kg)	15.7 (2.4–40.2)	19.3 (1.57–47.0)
Breed	12 Mixed breed, 9 Pitbull types, 2 Miniature Pinschers, 2 Shih Tzus and 11 other pedigree breeds	13 Mixed breed, 2 Labrador Retrievers, 2 Chihuahuas, 3 other pedigree breeds

Data are median (range)

Cronbach’s alpha for the full sedation scale was excellent (alpha =0.89). Inter-rater reliability was very good (ICC_single_ = 0.95; ICC_average_ = 0.99). The higher value for ICC_average_ reflects the improved reliability of multiple observers.

**Fig. 1:**
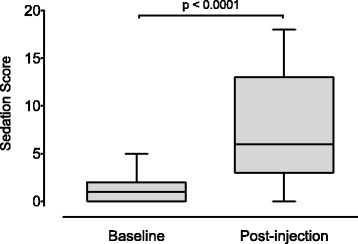
Sedation scores recorded before (baseline) and 15 min after injection of sedative drugs (post-injection). Box and whisker plots show median (central horizontal line), inter-quartile range (box boundaries) and Tukey whiskers

Sedation level increased in the majority of dogs following injection (*p* < 0.0001, median difference = 5, 95% CI [4 to 8] Fig. 1). Baseline scores ranged from 0 to 5 (median = 1). Post-injection scores varied greatly between individuals, ranging from 0 to 18 (median = 6).

Seven dogs (11.3%) did not show an increase in sedation score. These dogs received sedation via an intramuscular injection. Three of the dogs received acepromazine (0.03 mg/kg)-hydromorphone (0.05 mg/kg), two received dexmedetomidine (4.2–4.8 mcg/kg)-hydromorphone (0.05 mg/kg), one each received meperidine (2.9 mg/kg)-acepromazine (0.02 mg/kg) and morphine (0.5 mg/kg)-acepromazine (0.03 mg/kg).

**Fig. 2:**
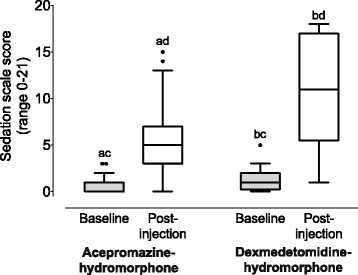
Sedation scores recorded before (baseline) and 15 min after injection (post-injection) of the two most common sedation protocols. Acepromazine-hydromorphone, *n* = 36. Dexmedetomidine-hydromorphone, *n* = 20. Box and whisker plots show median (central horizontal line), inter-quartile range (box boundaries) and Tukey whiskers. Identical letters indicate statistically significant differences for comparisons within (baseline versus post-injection) and between (baseline scores and post-injection scores) groups. See text for presentation of *p* values and 95% CI

The two most commonly used sedation protocols were dexmedetomidine (2.5–9.9 mcg/kg)-hydromorphone (0.04–0.1 mg/kg, *n* = 20), and acepromazine (0.01–0.05 mg/kg)-hydromorphone (0.05–0.1 mg/kg, *n* = 36). In general, both drug combinations were effective at increasing levels of sedation (Fig. 2). Acepromazine-hydromorphone, baseline versus post-injection: *p* < 0.0001, median difference = 4.5, 95% CI (3 to 6). Dexmedetomidine-hydromorphone, baseline versus post-injection: *p* < 0.0001, median difference = 10.5, 95% CI (6 to 13). Baseline sedation levels were slightly higher in dogs given dexmedetomidine-hydromorphone than those receiving acepromazine-hydromorphone (*p* = 0.02, median difference = 1, 95% CI [0 to 1]). At post-injection, dogs receiving dexmedetomidine-hydromorphone were significantly more sedated than dogs given acepromazine-hydromorphone (*p* = 0.001, median difference = 6, 95% CI [2 to 9]).

Post-injection, seven dogs (11.3%) had no jaw tone and did not swallow or show tongue movement when stimulated with a tongue depressor placed at the base of the tongue. Five of these dogs were given dexmedetomidine (3.9–9.9 mcg/kg)-hydromorphone (0.05–0.1 mg/kg), and two were given dexmedetomidine (9.6–14.4 mcg/kg)-hydromorphone (0.05–0.1 mg/kg)-ketamine (2.9–3.0 mg/kg). The sedation scores of the dogs without a swallow reflex were 17–18 out of 21 (dexmedetomidine-hydromorphone) and 18 out of 21 (dexmedetomidine-hydromorphone-ketamine). All dogs that received acepromazine-hydromorphone swallowed or showed tongue movement in response to stimulation.

No dogs showed an absence of palpebral reflex at 15 min post-sedation. Of the dogs that lost jaw tone/swallow reflex, four had a slow palpebral reflex but with a full corneal sweep, and three maintained a brisk palpebral reflex.

The psychometric properties of the sedation scale were considered acceptable, with a total weighted score of 14.8/20, when graded against a recently established set of criteria for assessing psychometric properties of health measurement scales (Appendix 1) [11, 15]. The distribution of scores by item was as follows: Scale development; 0.8/2, Reliability; 6/6, Validity; 6/8, Feasibility; 2/2.

### Abbreviated scale

**Fig. 3:**
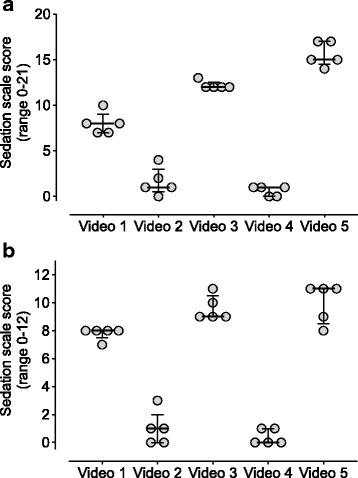
Sedation scale scores for the full (**a**) and abbreviated (**b**) scale, showing scores from 5 representative videos (15 videos scored in total). Same 5 videos shown in (**a** and **b**). Each circle is the score assigned by an individual rater. Medians and inter-quartile ranges are indicated by horizontal lines and whiskers. Inter-rater reliability was very good for both the full scale (**a**; ICC_single_ = 0.95) and abbreviated scale (**b**; ICC_single_ = 0.94)

The abbreviated scale (removal of 3 items: palpebral reflex, jaw and tongue relaxation, and resistance when laid into lateral recumbency, Table 3) maintained excellent internal consistency (Cronbach’s alpha =0.84). Additionally, applying the abbreviated scale to edited video-recordings maintained very good inter-rater reliability (ICC_single_ = 0.94; ICC_average_ = 0.99; Fig. 3). The time to complete assessment was approximately 3 times shorter for the abbreviated scale (40 [29–70] seconds) than the full scale (128 [63–206] seconds).

**Table 3:** Demographic data for dogs included in the 15 videos scored by the animal health technicians and interns

Number of dogs	14
Age (years)	4 (0.17–10)
Sex	5 males, 4 females, 3 female spayed, 2 male neutered
Mass (kg)	7.8 (2.4–28.8)
Breed	3 Mixed breed, 3 Pitbull types, 2 Pomeranian, Pug, Chihuahua, Pyrenean Mountain Dog, English Bulldog, Bichon Frise, Cocker Spaniel
Sedation	Baseline (*n* = 4), acepromazine-hydromorphone (*n* = 7), dexmedetomidine-hydromorphone-ketamine (*n* = 2), acepromazine (*n* = 1), dexmedetomdine-butorphanol (*n* = 1)

Data are median (range)

Both full and abbreviated scale scores showed a high degree of correlation between the primary (MW) and untrained observers (Additional file 1: Figure S1). The r value for the full sedation scale was 0.977 (95% CI 0.93 to 0.99, *p* < 0.0001) and for the abbreviated scale it was 0.990 (95% CI 0.97 to 1.0, *p* < 0.0001).

## Discussion

These data show that: 1. the sedation scale studied shows excellent internal consistency and is able to discriminate between different levels of sedation, 2. both the full and abbreviated scale versions show very good inter-rater reliability when applied by untrained raters, 3. high levels of sedation may be associated with loss of the swallow reflex, potentially increasing the risk of aspiration.

Internal consistency was excellent for both the full and abbreviated versions of the sedation scale. Internal consistency reflects the closeness of the relationship between scale items, the extent to which they measure the same general outcome (e.g. sedation) [16]. Together with an ability to discriminate between levels of sedation, this shows that the studied scale measures, and appears to be sensitive to, changes in sedation.

Combining an alpha_2_-adrenergic receptor agonist with an opioid increases the depth and quality of sedation compared with an alpha_2_-adrenergic receptor agonist alone [3, 10]. These data reflect the findings of Grint et al. [3], where the same sedation scale was used to assess the effect of medetomidine and pethidine and the resultant scores (approximately 15/21) were very similar to those reported here.

Using a 15 min observation period successfully exploited pharmacodynamic differences between dexmedetomidine and acepromazine, allowing discrimination between the sedative effects of each drug. Peak sedation occurs between 10 and 20 min after administering (IM or IV) dexmedetomidine [5, 6, 8], but approximately 30 min after administering acepromazine (IM) [17–19].

Scores from this study provide validation evidence for the use of the sedation scale and sedation protocols within our clinical context. That is, there is evidence to support that the scales measure what they are designed to measure, and scores from raters are able to detect changes in sedation between different sedation protocols and when compared with baseline.

Assessing and reporting inter-rater reliability is crucial when multiple raters are involved in data collection [16, 20–22]. Doing so confirms agreement between raters, providing confidence that assigned scores are comparable. Several studies have shown rater reliability to vary considerably with experience (specialists versus trainees, changes over time) and between raters with similar training [23, 24]. This inter-rater variability may negatively affect study outcomes and clinical case management by introducing data variability and reducing power, and influence the accuracy of diagnoses [25–27].

The inter-rater reliability of the studied scale (full and abbreviated versions) was very good even though raters were untrained and unfamiliar with the scale prior to participating in the study. The observed consistency between raters suggests that scale-specific training may not be required, and that sedation scores may be comparable across studies. This shows promise for its application in research and clinical environments.

Seven dogs had no jaw tone, tongue movement or swallowing at the post-injection assessment. Those dogs received either dexmedetomidine-hydromorphone or dexmedetomidine-hydromorphone-ketamine. This was an unexpected and concerning finding as it indicates a potential loss of the cough reflex and consequent inability to protect the airway. Though the cough reflex was not assessed directly by attempting orotracheal intubation, the ability to perform orotrachal intubation has been reported in dogs receiving an alpha_2_-adrenergic agonist alone or in combination with an opioid [2, 10]. Twenty-five percent of dogs (5/20) receiving IM medetomidine (40 mcg/kg) could be intubated approximately 20 min after injection [2] and the addition of fentanyl (2 mcg/kg IV) 16–18 min after IM medetomidine (20 or 40 mcg/kg) allowed all dogs (*n* = 6) to be intubated [10]. Furthermore, dogs without tongue movement and swallowing maintained a palpebral reflex, indicating that presence of a palpebral reflex may be a poor predictor of a maintained cough reflex.

Combining dexmedetomidine with a potent mu receptor agonist, such as hydromorphone, is a common protocol for sedation and premedication in dogs. In light of these and previous findings, the potential loss of protective airway reflexes should be considered when high levels of sedation are observed. The potent sedation created by medetomidine and dexmedetomidine exhibit a dose dependent duration [5]. Sedation lasting up to 240 min has been reported [4–6, 28, 29]. This makes a strong argument for pharmacological antagonism of medetomidine or dexmedetomidine with atipamezole once the procedure is completed.

A recent study in cats suggests that when multimodal analgesia is employed, antagonism of dexmedetomidine does not compromise analgesia [21]. Furthermore, a rapid return to normal function supports the concept of “enhanced recovery after surgery” whereby post-procedural morbidity and mortality is reduced through optimizing multiple aspects of patient care, including a rapid, smooth, pain-free recovery [21, 30].

The abbreviated scale was developed to shorten the assessment time, thereby improving feasibility and minimizing risk to personnel performing the assessment. Our preliminary assessment of the abbreviated scale shows it performs well, but further work is required to assess its ability to discriminate between levels of sedation.

The sedation scale performed well when evaluated against predetermined criteria established for evaluating the psychometric properties of health measurement scales in humans [11, 15]. To our knowledge, this is the first report of a formal evaluation of a health measurement scale using this scoring system in veterinary medicine. While acceptable, the performance of the scale against the scoring system indicates that further work is required. Practical implementation (feasibility) of the scale in a clinical setting, with diverse raters, remains to be fully determined. Convergent validation, comparison of the scale against an alternative measure of sedation, is possible though potentially challenging, as typical measures might include a form of electroencephalography. Finally, while scale items have clearly been adjusted through numerous iterations by experimenters with considerable experience, there is an absence of explicit description of how the scale items came to be selected.

This study was designed to assess the validity and reliability of a sedation scale, rather than compare different sedation protocols. Hence, routes of administration were not controlled and the time between injection and beginning the scheduled procedure was determined by the participating clinics. The latter restricted the post-injection assessment to 15 min. In combination with different routes of injection, it is likely that peak sedation was not achieved in many dogs that received acepromazine.

It was not possible for the primary observer (MW) to be blinded to treatment or time point. However the significant correlation with scores from the untrained observers for both the full and abbreviated scale indicates that scoring was unbiased.

The ability of an assessment scale to perform in varied settings reflects generalizability, a feature that can only be assessed by reporting psychometric properties (validity and reliability) in these situations [20, 22]. Therefore the population studied (age, breed, sex, mass) and setting (sedation protocols, raters, physical environment) should not be taken as a guarantee of scale performance in all settings [15, 16, 20, 22]. However, the diversity of breeds, sedation protocols and use of untrained raters indicates that the scale is likely to perform well in a range of settings and that data collected with the same scale could be compared across studies.

## Conclusions

Scores from the sedation scale provided evidence for excellent internal consistency and very good inter-rater reliability and these characteristics were maintained with the abbreviated scale. It was robust to the heterogenous population and study parameters indicating that it has good generalizability to a range of settings, potentially allowing a direct comparison of data between studies.

## Appendix 1

Brief description of data: Recently established criteria used to assess the psychometric performance of health assessment scales. The sedation scale studied is receives a total weighted score of 14.8/20, meeting the criteria for having acceptable psychometric properties (≥ 12/20) [11, 15]. The distribution of scores by item is: Scale development; 0.8/2, Reliability; 6/6, Validity; 6/8, Feasibility; 2/2. Table reproduced with the written permission of the publisher, Wolters Kluwer Health, Inc., through the Copyright Clearance Centre.

## Additional file

Additional file 1: Brief description of data: Correlation between sedation scores assigned by primary observer (MW) and untrained observers for the full sedation scale (A) and abbreviated sedation scale (B). (PDF 134 kb)

## Abbreviations

CI: Confidence interval
ICC: Intraclass correlation coefficient
