# Supplementary material for: Sedation levels in dogs: a validation study
Source: BMC Vet Res. 2017 Apr 18;13:110. doi: 10.1186/s12917-017-1027-2 (PMC5395740; doi:10.1186/s12917-017-1027-2)
Supplement: Additional file 1: — Brief description of data: Correlation between sedation scores assigned by primary observer (MW) and untrained observers for the full sedation scale (A) and abbreviated sedation scale (B). (PDF 134 kb) [file 12917_2017_1027_MOESM1_ESM.pdf]

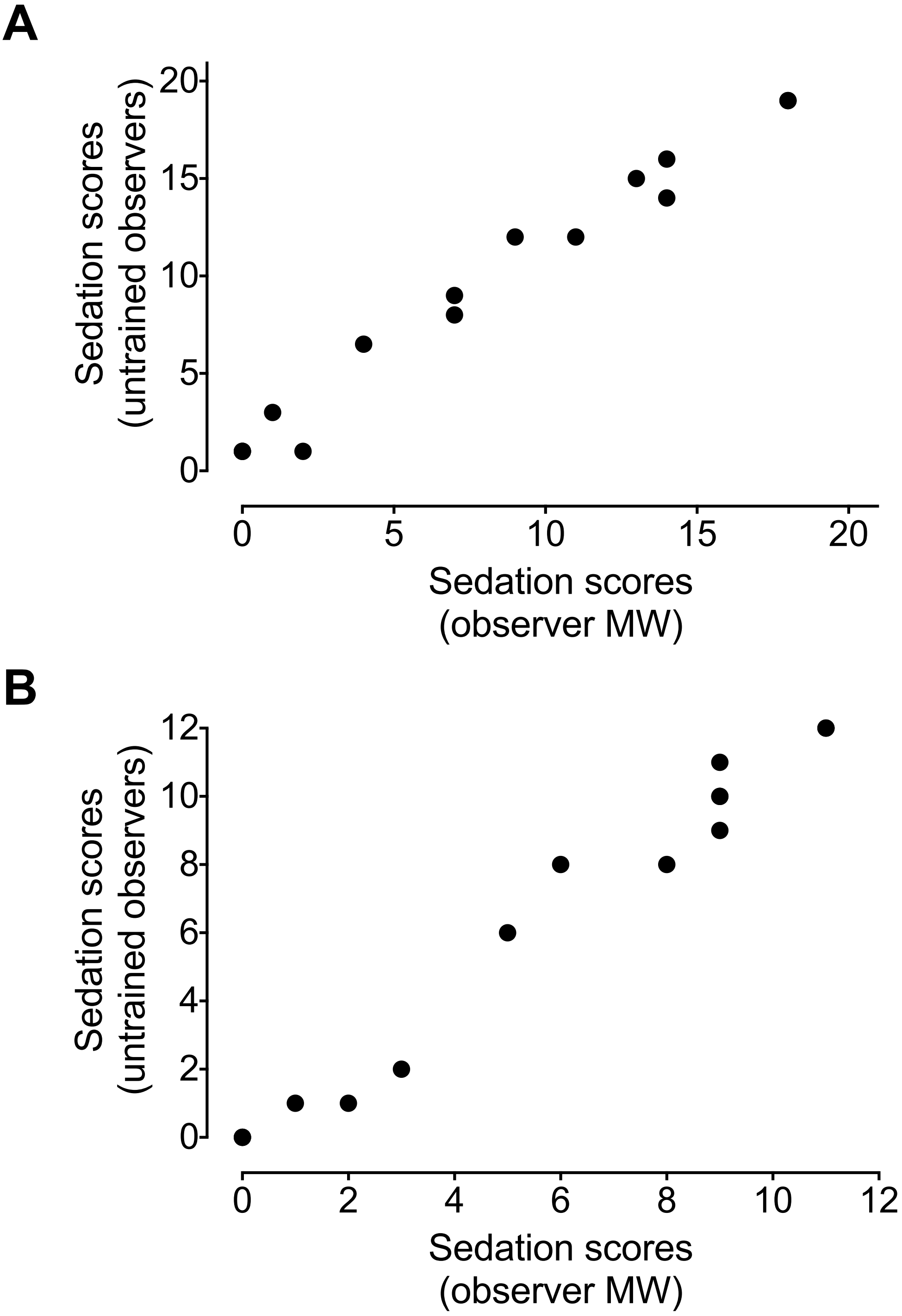

Additional file 1: Figure S1: correlation between sedation scores assigned by primary observer (MW) and untrained observers for the full sedation scale (A) and abbreviated sedation scale (B).
